# Meta-analysis of integrated ChIP-seq and transcriptome data revealed genomic regions affected by estrogen receptor alpha in breast cancer

**DOI:** 10.1186/s12920-023-01655-z

**Published:** 2023-09-15

**Authors:** Zeynab Piryaei, Zahra Salehi, Esmaeil Ebrahimie, Mansour Ebrahimi, Kaveh Kavousi

**Affiliations:** 1 Department of Bioinformatics, Kish International Campus University of Tehran, Kish, Iran; 2 Laboratory of Complex Biological Systems and Bioinformatics (CBB), Department of Bioinformatics, Institute of Biochemistry and Biophysics (IBB), University of Tehran, Tehran, Iran; 3 Hematology-Oncology and Stem Cell Transplantation Research Center, Tehran University of Medical Sciences, Tehran, Iran; 4 Genomics Research Platform, School of Agriculture, Biomedicine and Environment, La Trobe University, Melbourne, VIC Australia; 5 School of Animal and Veterinary Sciences, The University of Adelaide, Adelaide, South Australia Australia

**Keywords:** Meta-analysis, ChIP-seq, RNA-seq, Breast cancer, Estrogen receptor-positive, MCF7/T47D cell lines

## Abstract

**Background:**

The largest group of patients with breast cancer are estrogen receptor-positive (ER^+^) type. The estrogen receptor acts as a transcription factor and triggers cell proliferation and differentiation. Hence, investigating ER-DNA interaction genomic regions can help identify genes directly regulated by ER and understand the mechanism of ER action in cancer progression.

**Methods:**

In the present study, we employed a workflow to do a *meta-analysis* of ChIP-seq data of ER^+^ cell lines stimulated with 10 nM and 100 nM of E2. All publicly available data sets were re-analyzed with the same platform. Then, the known and unknown batch effects were removed. Finally, the *meta-analysis* was performed to obtain meta-differentially bound sites in estrogen-treated MCF7 cell lines compared to vehicles (as control). Also, the *meta-analysis* results were compared with the results of T47D cell lines for more precision. Enrichment analyses were also employed to find the functional importance of common meta-differentially bound sites and associated genes among both cell lines.

**Results:**

Remarkably, *POU5F1B, ZNF662, ZNF442, KIN, ZNF410*, and *SGSM2* transcription factors were recognized in the *meta-analysis* but not in individual studies. Enrichment of the meta-differentially bound sites resulted in the candidacy of pathways not previously reported in breast cancer. *PCGF2, HNF1B, and ZBED6* transcription factors were also predicted through the enrichment analysis of associated genes. In addition, comparing the *meta-analysis* results of both ChIP-seq and RNA-seq data showed that many transcription factors affected by ER were up-regulated.

**Conclusion:**

The *meta-analysis* of ChIP-seq data of estrogen-treated MCF7 cell line leads to the identification of new binding sites of ER that have not been previously reported. Also, enrichment of the meta-differentially bound sites and their associated genes revealed new terms and pathways involved in the development of breast cancer which should be examined in future in vitro and in vivo studies.

**Supplementary Information:**

The online version contains supplementary material available at 10.1186/s12920-023-01655-z.

## Introduction

Breast cancer (BC) is the world’s most widespread cancer among women [1]. The majority of BC patients are estrogen receptor-positive (ER^+^), i.e., Cancer cells have estrogen receptors (ESR1) [2, 3]. When the ER interacts with estrogen, it acts as a transcription factor (TF) and triggers cell proliferation and differentiation [4]. Hence, finding ER-DNA interaction genomic regions can help identify genes that are regulated by ER. Specifically, investigating these genomic regions can help to understand ER modes of action in cancer progression.

Chromatin immunoprecipitation sequencing (ChIP-seq), as a next-generation sequencing technique, is applied to find the transcription factor binding sites (TFBSs) [5]. In the present era, several ChIP-seq datasets have been generated to find differentially bound sites (DBSs) in BC patients [6–8]. Each study has results unique or commonalities with other related studies, which could be due to known and unknown batch effects [6–8]. On the other hand, gathering relevant studies and performing *meta-analysis* supplies more exact results [9]. Few studies have been performed on the *meta-analysis* of ChIP-seq data. A *meta-analysis* study was conducted by Kolmykov and colleagues on ChIP-seq datasets through the rank aggregation approach, and the significant TFBSs in BC were identified [10]. Some complexities with ChIP-seq data should be considered for *meta-analysis*, including: (1) it is necessary to consider the same cell line, dose, and treatment period (known batch effects), (2) selecting the appropriate treatment period, i.e., after which treatment period, treated and untreated samples show the most differentiation, (3) removing the effects of differences laboratory conditions (unknown batch effects) seems to be vitally important to obtain more precise *meta-analysis* results, (4) since each study may have been analyzed with different platforms and genomic references, it is necessary to re-analyze the samples with the same tools and references, and (5) it is noteworthy that even if the same platform is applied, a different number of peaks and regions are obtained for each sample. Therefore after combining the datasets, they must be prepared to obtain the same number, regions, and scores of TFBSs for all samples. Next, the unknown batch effects must be removed before *meta-analysis*. Despite the necessities mentioned above, no *meta-analysis* study has already been performed on integrated TFBSs to obtain significant binding sites in BC.

In the current study, to obtain meta-differentially bound sites in estrogen receptor-positive breast cancer cell lines, we employed an innovative workflow for the meta-analysis of ChIP-seq data. Given the ER's essential role in BC, MCF7 and T47D cell lines stimulated with E2 were collected to find genomic regions directly interacting with ER. MCF7 and T47D cell lines, representing luminal A subtype, are extensively utilized as experimental models in breast cancer research, particularly for the study of hormone-dependent BC. These two ER + BC cell lines serve as valuable tools in understanding the molecular mechanisms and exploring potential therapeutic interventions for BC, particularly luminal A subtype. In the MCF7 cell line, concerning the same cell line, dose, and treatment period (removing known batch effects), public ChIP-seq datasets were selected and re-analyzed. Next, samples were integrated, and the same number, regions, and scores of TFBSs were obtained in doses of 10 nM and 100 nM separately. Finally, unknown batch effects were removed, and several ChIP-seq datasets were meta-analyzed based on TFBSs scores. Hence, we take advantage of the term meta-differentially bound sites (meta-DBSs) for the first time. For more precision, the *meta-analysis* results were compared with the results of T47D cell lines, and the intersection of both cell lines was obtained. By enriching the shared meta-DBSs and their associated genes, TFs and pathways that had not been previously reported in BC were identified. Moreover, the results of the ChIP-seq data *meta-analysis* were confirmed by comparing the results of the RNA-seq data *meta-analysis*.

## Materials and methods

**Fig. 1:**
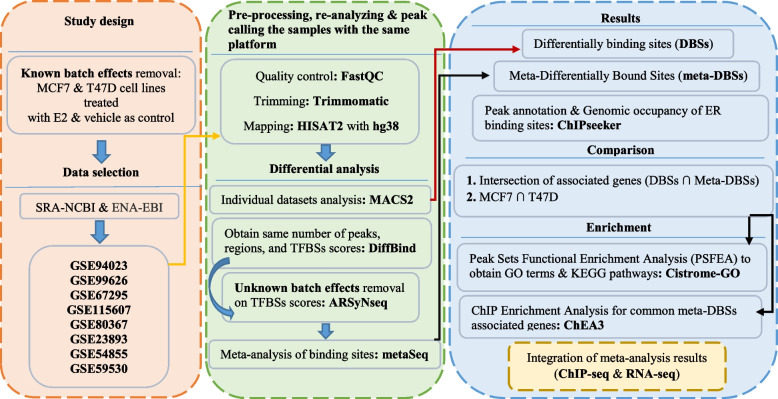
An overview of the *meta-analysis* steps performed in the present study. ChIP-seq datasets were retrieved from SRA-NCBI and ENA-EBI databases. Pre-processing and re-analyzing steps of datasets were conducted. Then, batch effects removal and *meta-analysis* were performed. Subsequently, peak set functional enrichment analysis (PSFEA) and ChIP Enrichment Analysis for meta-DBSs-associated common genes were performed using the Cistrome-GO database and ChEA3 database, respectively. The packages and methods employed are in bold form. *TFBSs* Transcription factor binding sites, *DBSs* Differentially bound sites, *Meta-DBSs* Meta-differentially bound sites

After collecting data from SRA-NCBI [11] and ENA-EBI [12] databases, nine MCF7 and T47D cell line-associated ER alpha-ChIP-seq datasets from eight studies were selected. Differential analysis for each dataset was performed, and DBSs were obtained following data pre-processing and mapping. Next, the *meta-analysis* was performed, and meta-DBSs were identified in the MCF7 cell line treated with 10 nM and 100 nM E2 separately. Then, Peak Annotation was applied for DBSs and meta-DBSs, and the results of both cell lines were compared. Peak set functional enrichment analysis (PSFEA) and ChIP enrichment analysis (ChEA) were implemented for the common meta-DBSs and their associated genes, respectively. Also, the *meta-analysis* results of both ChIP-seq and RNA-seq data were compared. The general steps of the present study are summarized in Fig. 1.

### Data collection

**Fig. 2:**
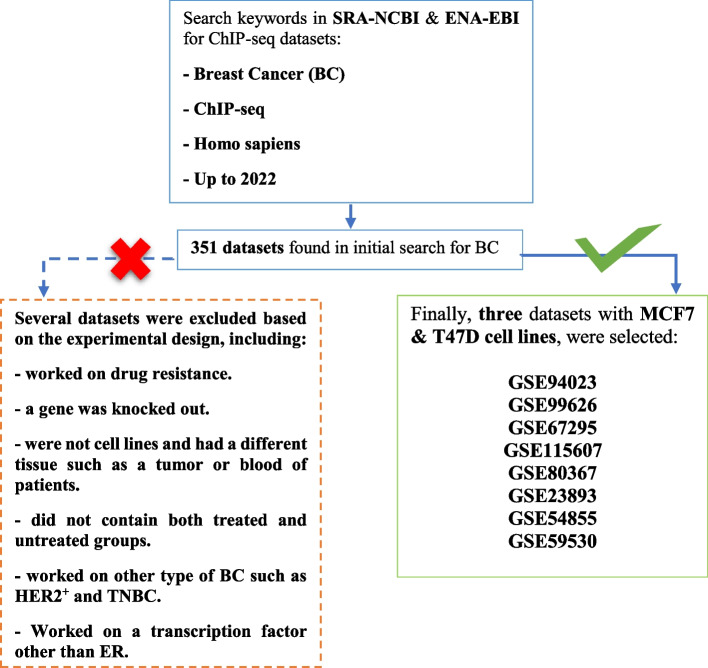
The flowchart to select datasets. A total of 351 datasets from SRA-NCBI and ENA-EBI were evaluated. Finally, based on the four criteria described, eight studies on MCF7 and T47D cell lines treated with E2 were used in the present study

A comprehensive search was conducted on SRA-NCBI [11] and ENA-EBI [12] databases to find ChIP-seq datasets in BC (Fig. 2). Some criteria were considered in the selection of the ChIP-seq datasets: (1) association with ER^+^ cell lines; MCF7 and T47D cell lines, (2) availability of both treated with vehicles (as controls) and treated with E2 samples, (3) datasets without any knocked out genes, and (4) datasets with the same and appropriate dose and period of treatment. Consequently, GSE94023, GSE99626, GSE67295, GSE115607, GSE80367, GSE23893, GSE54855, and GSE59530, including MCF7 and T47D cell lines stimulated with 10 nM and 100 nM E2 for 40 or 45 min were selected. The details of the datasets are described in Table 1.

**Table 1:** Characteristics of the selected ER-ChIP-seq datasets and their experimental design

**Accession number**	Cell lines	Dose (nM) of E2	Inputs	Samples treated with vehicles as controls	Samples treated with E2	chip antibody	Type of vehicle treatment	Experiment
GSE94023	MCF7	10	-	GSM2467220	GSM2467224	F3A6 (to ERalpha, Gronemeyer)	mock	Dzida,…et al. [7]
GSE99626			GSM2125164 (Vehicle_Input) GSM2125165 (E2_Input)	GSM2648921	GSM2648922	anti-ER (SC biotech HC-20, cat# SC-543, lot# L1911)	ETOH	Singhal,…et al. [17]
GSE67295			-	GSM1643946 (rep1) GSM1643947 (rep2)	GSM1643948 (rep1) GSM1643949 (rep2)	ERa (Vendor: Santa Cruz, cat# sc-543, lot# C2114)	DMSO	Stender,…et al. [8]
GSE115607			GSM3184881	GSM3184871	GSM3184880	ERa (Santa Cruz, sc-543, lot F1716)	DMSO	Puyang,…et al. [6]
GSE80367	T47D		GSM2112811 (Vehicle_Input) GSM2112812 (E2_Input)	GSM2112796	GSM2112798	anti-ER (SC biotech HC-20)	ETOH	Singhal,…et al. [18]
GSE23893	T47D	100	GSM589247	GSM589238	GSM589239	ERα (sc-543, Santa-Cruz)	ETOH	Kong,…et al. [19]
GSE23893	MCF7		GSM589244	GSM589236	GSM589237	ERα (sc-543, Santa-Cruz)	ETOH	*Kong,…*et al*.* [19]
GSE54855			GSM1325252	GSM1325246	GSM1325250	ER (Santa Cruz, sc-542)	mock	Guertin,…et al. [20]
GSE59530			GSM1534712 (Vehicle_Input_rep1) GSM1534713 (Vehicle_Input_rep2) GSM1534714 (E2_Input_rep1) GSM1534715 (E2_Input_rep2)	GSM1534720 (rep1) GSM1534721 (rep2)	GSM1534722 (rep1) GSM1534723 (rep2)	ER alpha (rabbit polyclonal generated in the Kraus Lab)	unknown	Franco,…et al. [21]

*nM* Nanomolar, *ETOH* ethanol

### Pre-processing and data analysis

The selected ChIP-seq datasets were re-analyzed under the Galaxy platform (https://galaxyproject.org) [13] to homogenize studies. The data quality control and trimming were performed using FastQC (version 0.11.5) [14] and Trimmomatic (version 0.38) [15], respectively. The human reference genome (hg38) was utilized for data mapping by HISAT2 (version 2.1.0) [16].

#### Analysis of individual datasets to identify DBSs

Within each individual study, DBSs were identified in MCF7 and T47D cell lines treated with E2 compared to treated with vehicle (as control) through MACS2 [22] (version 2.1.1.20160309.6) (-log10 (^q−value^) > 2; fold-enrichment > 2). Then, the ChIPseeker (version 1.26.2) [23] package was applied for peak annotation. The identified DBSs associated genes of individual studies were further utilized to compare with the results of the ChIP-seq *meta-analysis*. During peak calling with MACS2, the input samples were also considered to reduce noise. The Blacklist regions were also considered to improve the signal-to-noise ratio. The maximum tags were set to keep one tag at the same location (–keep-dup = 1). In the individual dataset analysis, replicates were combined using the rmspc (Multiple Sample Peak Calling) package [24].

Typically, analysis of treatment, vehicle, and input samples on ChIP-seq data is performed in the following steps:

- A. ER-ChIP treated with E2 vs. input = corrected ER-ChIP treated with E2.
- B. Vehicle vs. input (for vehicle) = corrected vehicle.
- C. Corrected ER-ChIP treated with E2 vs. corrected Vehicle = detection of enriched regions in ER-ChIP treatment E2 (using MACS2).

#### *Meta-analysis* to identify meta-DBSs

**Fig. 3:**
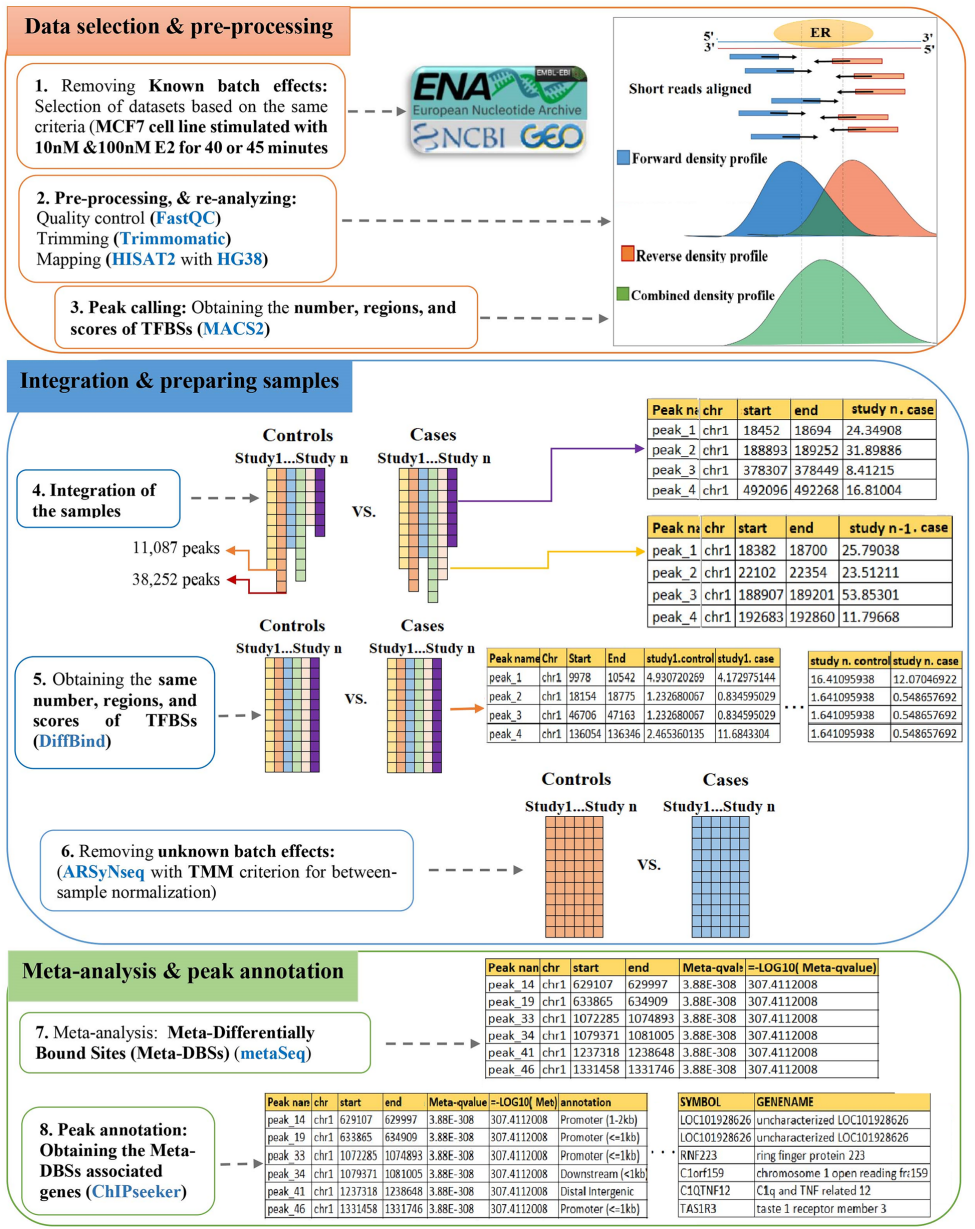
A workflow for integration and *meta-analysis* of ChIP-seq TFBSs scores from several studies. Datasets were selected based on the same criteria and re-analyzed with the same platform. Then samples were integrated, normalized, and meta-analyzed. This process was performed for MCF7 cell lines, and meta-DBSs were obtained. Next, meta-DBSs-associated genes were identified with peak annotation. The packages used in each step are marked in blue. *TFBSs* Transcription factor binding sites, *DBSs* Differentially bound sites, *Meta-DBSs* Meta-differentially bound sites

To find meta-DBSs, a *meta-analysis* workflow was utilized to compare cells of treated with vehicle versus treated with E2 at the TFBSs score levels (Fig. 3). First, the same number, regions, and scores of TFBSs were obtained utilizing the DiffBind package (Version 3.0.15) [25]. All replicates were considered as independent samples to produce the binding affinity matrix. Then, the ARSyNseq (ASCA Removal of Systematic Noise for sequencing data) method [26], implemented in the NOIseq package [26], was applied to remove unknown batch effects (Fig. 1). In ARSyNseq, the TMM (Trimmed Mean of M values) method [27] was utilized for between-sample normalization on TFBSs scores. Then, the metaSeq package [28] was employed on all samples to find meta-DBSs and set the statistical threshold as -log10 (^q−value^) > 2 to select meta-DBSs. Finally, the ChIPseeker package was utilized for peak annotation. All packages were implemented in R software. The parameters in the ARSyNseq and the metaSeq packages were set as follows:

mydata2corr1 = ARSyNseq (mydata2, factor = “batch”, batch = TRUE, norm = “tmm”) result <- meta.oneside.noiseq (cds, k=0.5, norm = “n”, replicates = “biological”, factor = flag1, conditions = c(1,0), studies = flag2)

### Peak set functional enrichment analysis and ChIP enrichment analysis

Gene ontology (GO) and pathway enrichment analyses were performed with Cistrome-GO (the updated version of 2019) (http://go.cistrome.org/) [29] to determine the functional importance of the identified meta-DBSs. For such analysis, we employed peak set functional enrichment analysis (PSFEA) term for the first time. The GO annotation was performed at three levels, including biological process (BP), molecular function (MF), and cellular component (CC). Furthermore, the Kyoto Encyclopedia of Genes and Genomes (KEGG) (the updated version of 2019) [30] enriched pathways were determined. The adjusted *P*-value ≤ 0.05 was applied to select significant GO terms and KEGG pathways. Also, a comprehensive ChIP Enrichment Analysis (ChEA) was performed on meta-DBSs-associated common genes using ChEA3 (the updated version of 2019) (https://amp.pharm.mssm.edu/ChEA3) [31], which contains six libraries, including Literature, Enrichr, ARCHS4—Coexpression, ENCODE, ReMap, and GTEx—Coexpression. Also, two overall results were obtained: (1) integrated_topRank, and (2) integrated_meanRank; in both of them TFs were ranked based on estimated scores in libraries. Adjusted *P*-value ≤ 0.05 was considered significant.

## Results

The *meta-analysis* was performed on the ChIP-seq data based on TFBSs scores to identify meta-DBSs. Here, known batch effects were first removed. Since the treatment period with E2 can affect the results, several treatment periods were investigated to select the appropriately treated cell lines. In the GSE94023 study, there were various periods of treatment with E2, including 0 (mock treated as control) and 5, 10, 20, 40, 80, 160, 320, 640, and 1280 min (cases). An appropriate sampling time reveals the maximum distinction between treated and untreated samples. Hence, a heat map correlation matrix was drawn for all samples. According to the matrix, the samples after 40 min showed the most significant difference compared to the control (see Additional file 1: Figure S1). Thus, samples treated with 10 nM and 100 nM E2 for 40 or 45 min were selected. The minimum peak length was considered equal to 150-bp as the significant peak cutoff.

### Identification of DBSs

After selecting and pre-processing data, DBSs were obtained from nine individual ChIP-seq datasets (Table 2; see Additional file 2: Tables S1-S9) and compared with the *meta-analysis* result*s*.

**Table 2:** Identified DBSs/ meta-DBSs through individual/ meta-analysis on eight studies of the MCF7 and T47D cell lines treated with 10 nM and 100 nM E2

**Analysis**	Accession number	Cell line	Treatment	Dose (nM)	DBSs or Meta-DBSs (n)	Associated genes (n)
**Individual analysis**	GSE94023	MCF7	E2	10	38,963	13,982
	GSE99626				15,372	9,013
	GSE67295				29,145	11,531
	GSE115607				21,939	11,020
	GSE80367	T47D			4,877	3,081
	GSE23893	T47D		100	1,882	1,320
	GSE23893	MCF7			11,155	6,272
	GSE54855				10,343	5,710
	GSE59530				29,915	10,817
**Meta-analysis**	GSE94023	MCF7		10	23,308	10,503
	GSE99626					
	GSE67295					
	GSE115607					
	GSE23893			100	7,796	4,591
	GSE54855					
	GSE59530					

*DBSs* differentially bound sites, *Meta-DBSs* meta-differentially bound sites, *nM* Nanomolar

### Identification of meta-DBSs

To identify meta-DBSs in MCF7 cell lines, datasets were integrated based on stimulation with 10 nM or 100 nM E2 separately. Next, DiffBind was applied to obtain the same number, regions, and scores of TFBSs (see Additional file 2: Tables S10 and S11). Following unknown batch effect removal (see Additional file 1: Figures S2-S5), ChIP-seq data were meta-analyzed, and the meta-DBSs (-log_10_ (^q−value^) > 2) were identified (see Additional file 2: Tables S12 and S13). The details of DBSs, meta-DBSs, and associated genes are described in Table 2.

### Shared significant TFBSs between meta-DBSs and DBSs

**Fig. 4:**
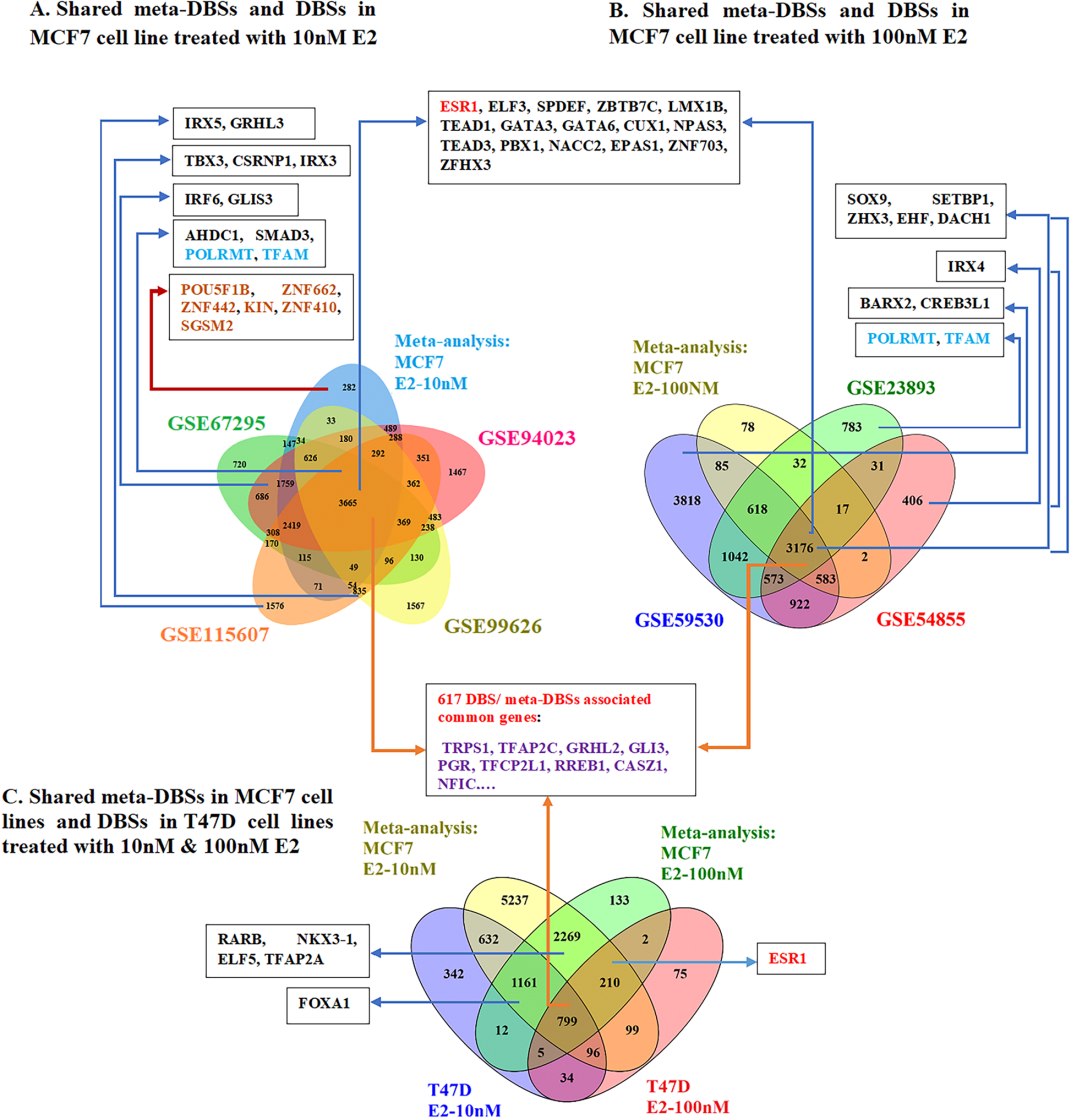
Shared meta-DBSs between *meta-analysis* and individual studies based on TFBSs associated genes in MCF7 and T47D cell lines treated with E2. Among the peaks associated genes, only TFs are displayed**.** Nine of these TFs, which are also among the top 50 TFs obtained from ChEA3, are shown in purple. The six TFs only identified through *meta-analysis* are shown in orange. The marked blue TFs are mitochondrial TFs. *TFs* Transcription factors, *DBSs* Differentially bound sites, *Meta-DBSs* Meta-differentially bound sites

Meta-DBSs were compared with DBSs obtained from individual datasets for more precision. Based on our findings, 617 genes were common among meta-DBSs- and DBSs- associated genes, 35 of which were TFs (Fig. 4). Remarkably, nine of those TFs were also identified among the top 50 TFs obtained from ChEA3 in Section 3.5.1 (Fig. 6). There were 282 genes associated with peaks identified through *meta-analysis* of MCF7 cell lines treated with 10 nM E2 but not in initial individual datasets. Six of them were *POU5F1B, ZNF662, ZNF442, KIN, ZNF410*, and *SGSM2* TFs (Fig. 4A). Moreover, most of the top 50 TFs were also identified among the *meta-analysis* results and some initial individual datasets (Figs. 4 and 6). *PCGF2, HNF1B, and ZBED6* TFs predicted by ChEA3 were not identified through *meta-analysis* or initial individual datasets.

### Genomic occupancy of ER binding sites

**Fig. 5:**
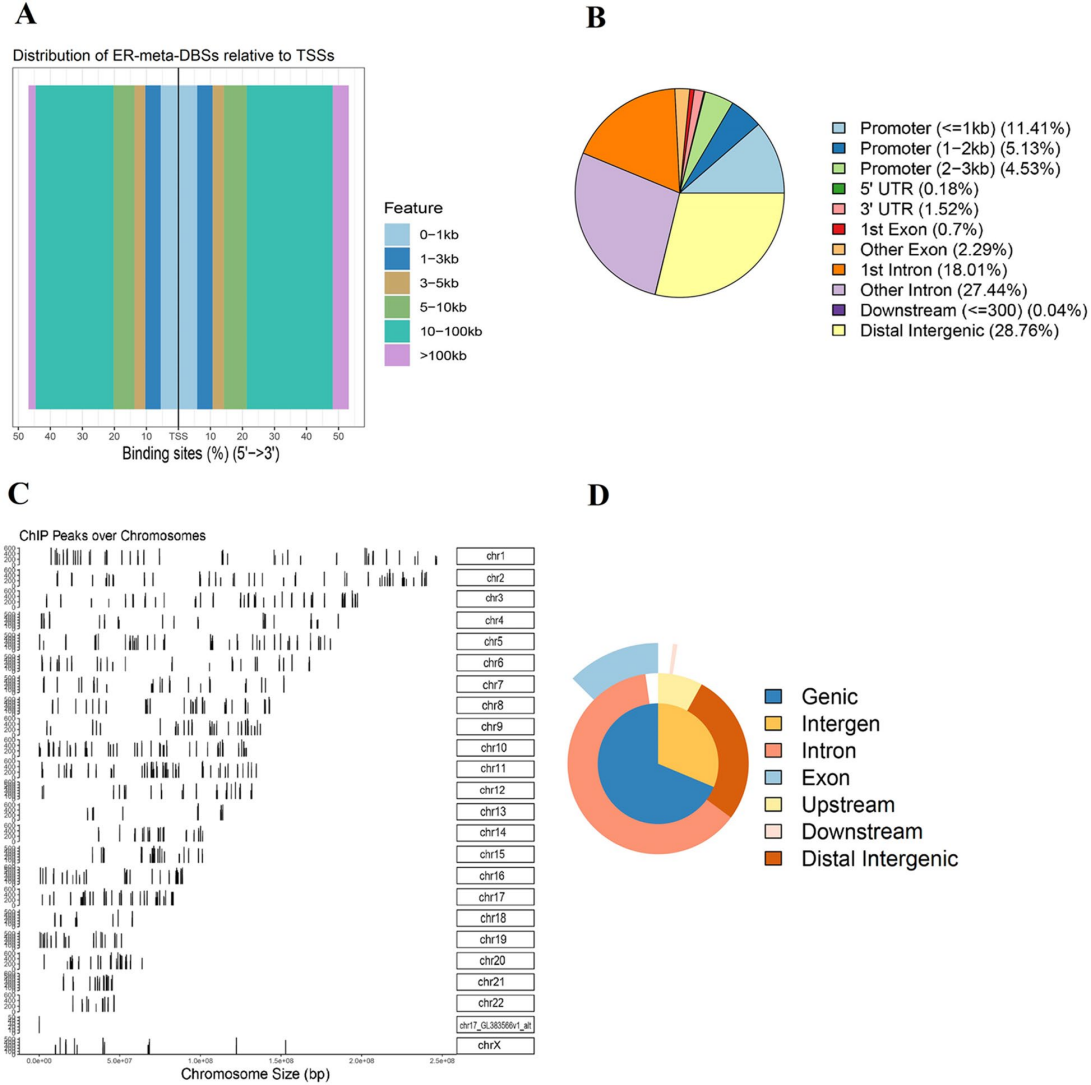
Genome-wide annotation of 7,308 meta-DBSs correlated with 617 common genes and response elements of ER between MCF7 and T47D cell lines treated with E2. **A** Distribution of ER-meta-DBSs relative to the nearest TSS across the human genome. **B** Pie plot of the ER-meta-DBSs percentages according to peak location across different genomic regions of the human genome. **C** Visualization of ER-meta-DBSs obtained from the UCSC genome browser (version hg38). **D** Venn pie of annotations and their overlap. *TSS* transcription start site

The genomic locations of 7,308 ER-meta-DBSs correlated with 617 common genes were annotated using the ChIPseeker. According to the genome-wide annotation of TFBSs, it was found that there were 1,534 binding sites located within 10 kb of a transcription start site (TSS) (Fig. 5A). 1,531 of these sites were situated in the proximal to the TSSs (promoter region). Also, peaks were 3,327 in introns, 2,104 in intergenic, 218 in exons, 13 in 5' untranslated regions (UTRs), three in TSSs (downstream region), and 112 in 3' UTRs (Fig. 5B and C). Because some of the annotations overlap, the complete annotations and their overlaps are illustrated in Fig. 5D. These results indicated that most ER binding sites are located in introns, followed by intergenic regions, promoter-TSSs, and exons to a lesser degree in 3' UTRs, TSSs, and 5' UTRs.

### Enrichment analysis

#### ChIP enrichment analysis

**Fig. 6:**
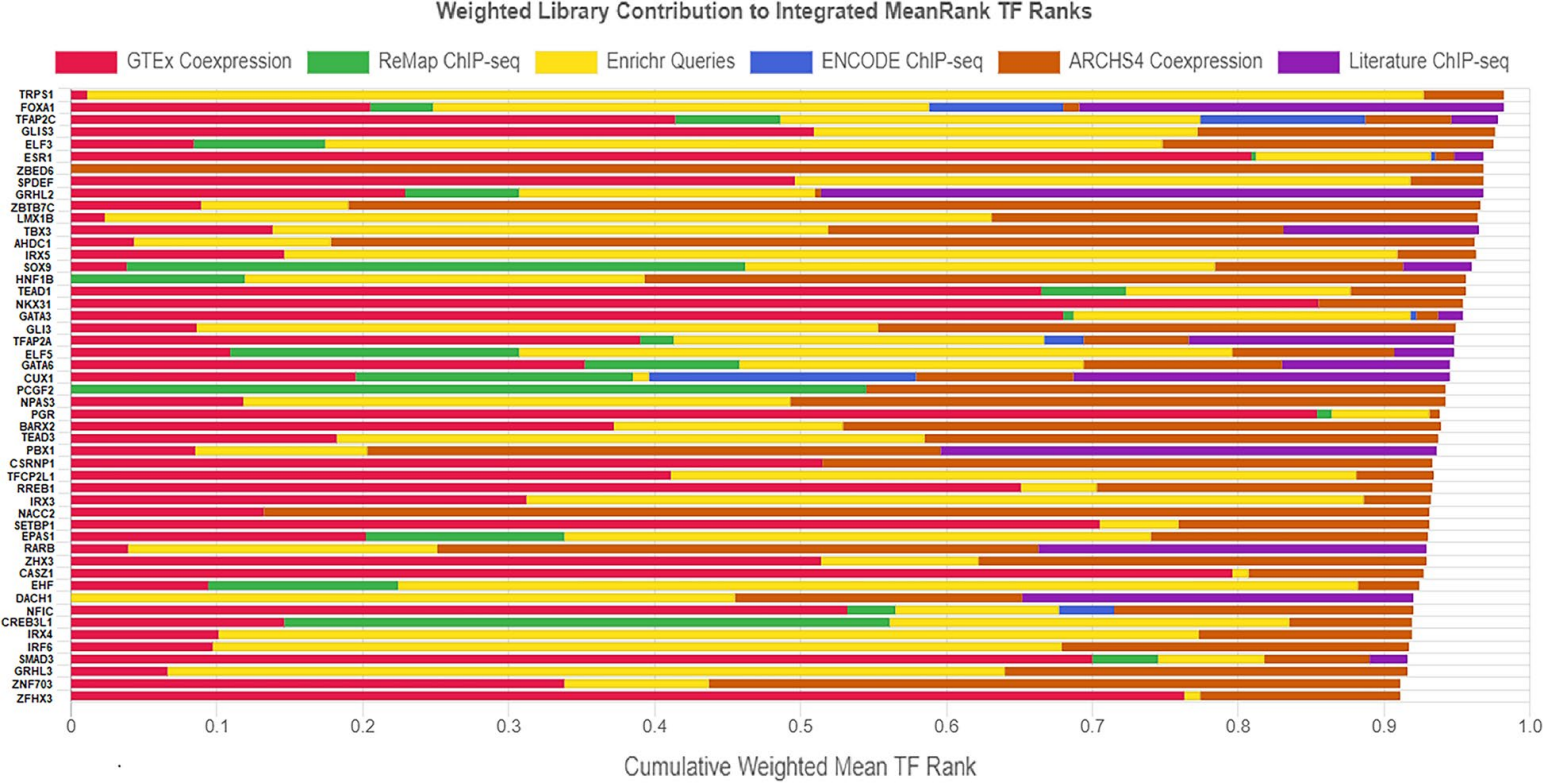
ChIP Enrichment Analysis (ChEA) for 617 meta-DBSs-associated common genes. The bar graph integrated_meanRank for the top 50 TFs using ChEA3 in MCF7 and T47D cell lines treated with E2. Each color represents a library, and each bar's length indicates the weight of that TF in each library. *TFs* transcription Factors

The 617 meta-DBSs-associated common genes were enriched using the ChEA3 database to predict significant TFs that regulate gene expression in breast cancer. The results of six libraries and two overall results are presented in Additional file 2: Tables S14-S21. In accordance with the integrated_meanRank (see Additional file 2: Table S21), 1632 TFs were ranked, and the top 50 TFs are shown in Fig. 6. Remarkably, *TRPS1, FOXA1, TFAP2C, GLIS3, ELF3, and ESR1* TFs have the highest rank score. Also, *PCGF2, HNF1B, and ZBED6* TFs were predicted as potential key regulators.

#### Peak set functional enrichment analysis

Peak set functional enrichment analysis, including GO and KEGG pathways for 7,308 meta-DBSs (correlated with common 617 genes) was performed. By enriching meta-DBSs and considering adjusted *P*-value ≤ 0.05, 94 BPs, 3 CCs, and 12 MFs for GO terms and 9 KEGG pathways were detected (see Additional file 2: Tables S22 and S23). GO terms regarding BPs were annotated in cornification (GO:0070268), tissue development (GO:0009888), regulation of anatomical structure morphogenesis (GO:0022603), anterior/posterior pattern specification (GO:0009952), phosphatidylcholine metabolic process (GO:0046470), negative regulation of transcription by RNA polymerase II (GO:0000122), response to metal ion (GO:0010038), negative regulation of RNA biosynthetic process (GO:1902679), embryonic organ morphogenesis (GO:0048562), and response to inorganic substance (GO:0010035).

**Fig. 7:**
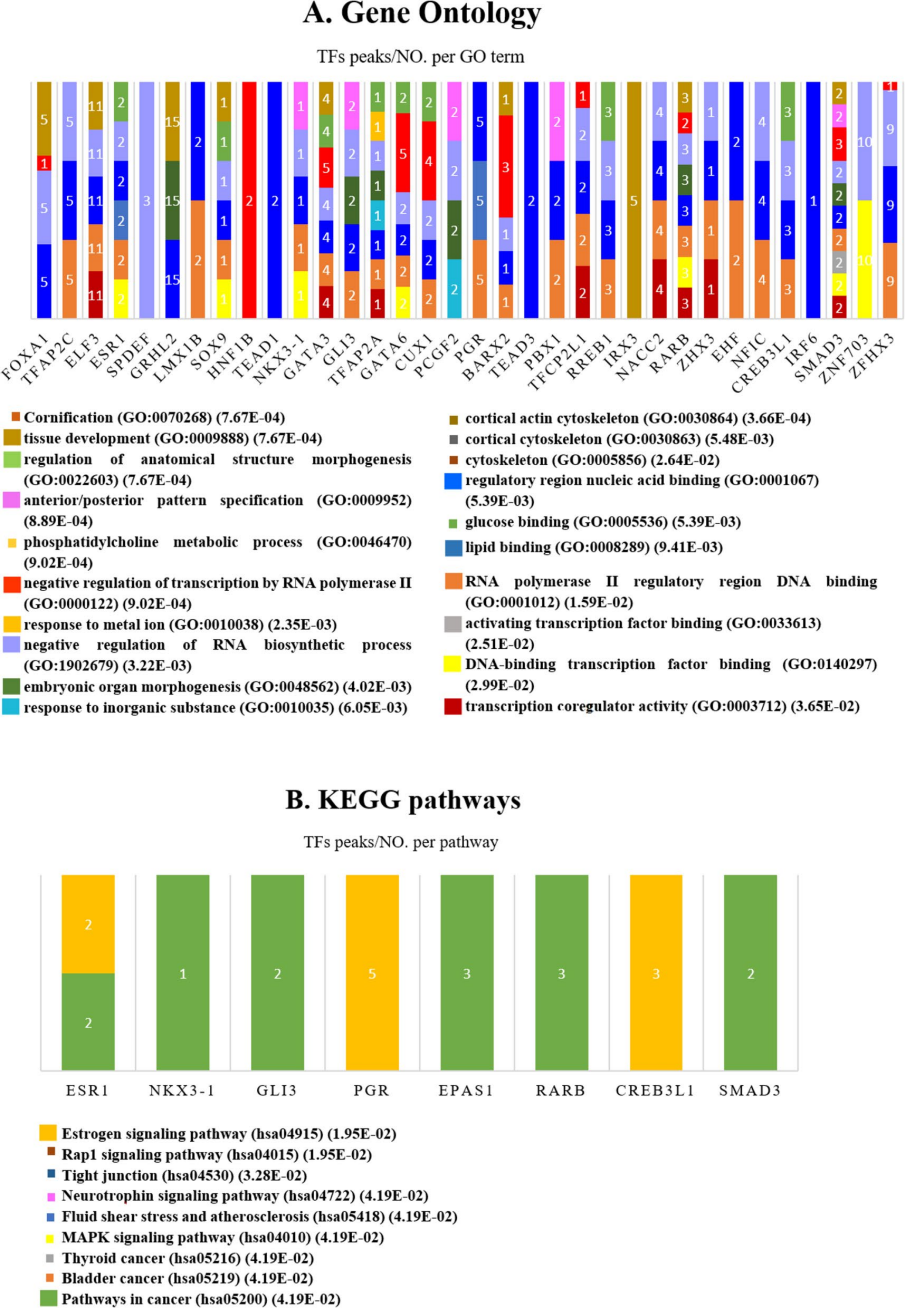
Peak set functional enrichment analysis (PSFEA) for 7,308 meta-DBSs, including GO and KEGG pathways. (**A**) The chart of the top 20 GO terms and (**B**) nine KEGG pathways was obtained from 7,308 meta-DBSs in MCF7 and T47D cell lines treated with E2. Also, TFs that enriched GO terms and KEGG pathways are shown along with the number of peaks. *TFs* Transcription factors, *DBSs* Differentially bound sites, *Meta-DBSs* Meta-differentially bound sites

Also, enriched CCs terms were linked to the cortical actin cytoskeleton (GO:0030864), cortical cytoskeleton (GO:0030863), and cytoskeleton (GO:0005856). Significant GO terms were found for MFs with regulatory region nucleic acid binding (GO:0001067), glucose binding (GO:0005536), lipid binding (GO:0008289), RNA polymerase II regulatory region DNA binding (GO:0001012), activating transcription factor binding (GO:0033613), DNA-binding transcription factor binding (GO:0140297), and transcription coregulator activity (GO:0003712) (Fig. 7A).

The nine enriched KEGG pathways included the Estrogen signaling pathway (hsa04915), Rap1 signaling pathway (hsa04015), Tight junction (hsa04530), Neurotrophin signaling pathway (hsa04722), Fluid shear stress and atherosclerosis (hsa05418), MAPK signaling pathway (hsa04010), Thyroid cancer (hsa05216), Bladder cancer (hsa05219), and Pathways in cancer (hsa05200) (Fig. 7B). Moreover, the top 50 TFs obtained from ChEA3 were examined. The TFs that enriched the Top 20 GO terms and nine KEGG pathways with the number of peaks in each route is shown in Fig. 7.

**Fig. 8:**
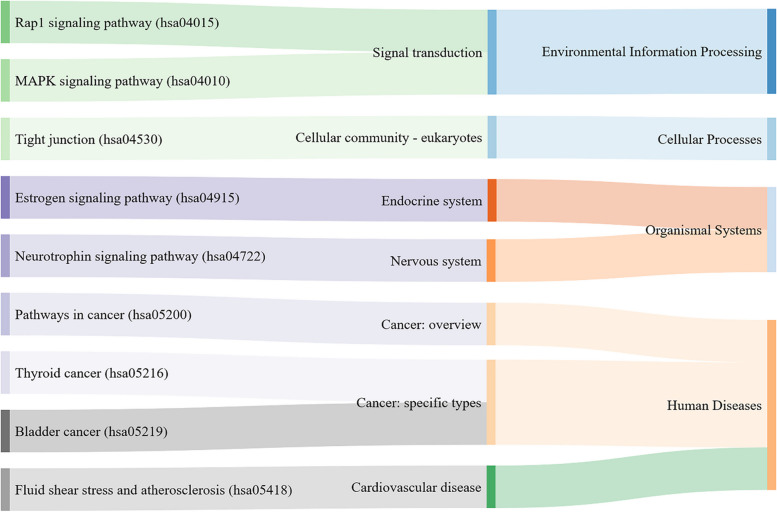
Sankey diagram for categories of KEGG pathways

A general classification of pathways obtained from the enrichment of meta-DBSs indicated that most of those pathways play a role in cancer, environmental information processing, and organismal systems (Fig. 8).

### Evaluation of ChIP-seq *meta-analysis *results along with RNA-seq *meta-analysis* results

Recently we performed a *meta-analysis* of RNA-seq data on both MCF7 and T47D cell lines treated with 10 nM E2 for 8 h [32]. We investigated the expression of the top 50 TFs predicted by ChEA3 (Table 3). Comparing *meta-analysis* results of ChIP-seq and RNA-seq data showed that many TFs were up-regulated in RNA-seq *meta-analysis* or individual datasets.

**Table 3:**
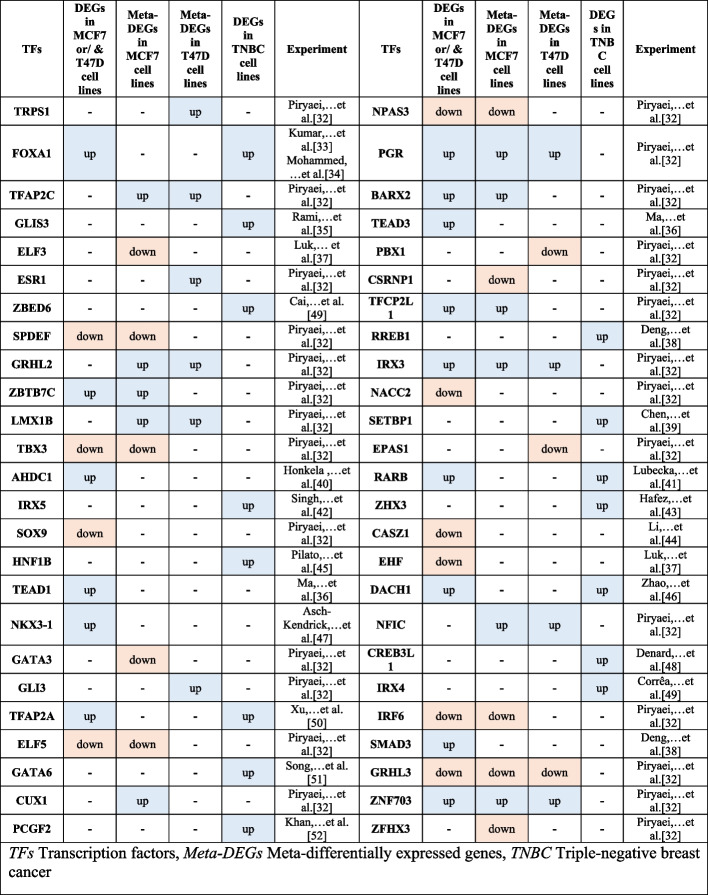
Differential express of top 50 TFs using ChEA3 for 617 meta-DBSs-associated common genes [32–52]

*TFs* Transcription factors, *Meta-DEGs* Meta-differentially expressed genes, *TNBC* Triple-negative breast cancer

## Discussion

Breast cancer is the most incident cancer among women worldwide. The largest group of patients with BC is ER^+^. The estrogen receptor plays the role of a transcription factor when exposed to estrogen and triggers cellular proliferation and differentiation [2, 3]. Hence, evaluating the expression of TFs directly affected by ER can help identify and understand ER action's mechanism in cancer progression. Remarkably, combining more samples and performing *meta-analysis* will produce more stringent results than single experiments [9]. To the best of our knowledge in the current study, for the first time, we applied the workflow for *meta-analysis* of ChIP-seq data based on TFBSs in the MCF7 cell lines treated with 10 nM and 100 nM E2. First, samples were integrated, and known and unknown batch effects were removed. Next, the *meta-analysis* approach was conducted on TFBSs scores, and meta-DBSs were obtained. Our results showed significant peaks (specifically for TFs affected by ER) identified through the *meta-analysis* but not found in individual studies. For the strict selection of meta-DBSs, MCF7 cell lines *meta-analysis* results were compared with the results of T47D cell lines and initial individual studies, and common genes and the associated meta-DBSs were determined. With the enrichment of meta-DBSs correlated with common genes, several pathways with presumably clinical importance in BC were identified. Meta-DBSs-associated common genes were also enriched, and TFs were identified that have been predicted as potential key regulators in the progression of BC. In addition, we compared the *meta-analysis* results of both ChIP-seq and RNA-seq data.

Some of the differentially expressed genes (DEGs) identification methods in the RNA-seq context, such as DEseq2 [53] and edgeR [54], are also exploited to discover DBSs. However, the NOIseq package [26, 28] performs relatively better for obtaining DEGs [28]. Moreover, packages were developed for *meta-analysis* of RNA-seq data based on those methods [28, 55, 56]. To find meta-DBSs, we utilized the metaSeq package [28], in which NOIseq has been used. Before performing the *meta-analysis*, we faced two problems: (1) none of the mentioned methods removed unknown batch effects, and (2) the number of peaks and their associated genomic start and end intervals were different among samples, and batch effects could not be removed. Therefore, sample preparation was required. To this end, we obtained the same number, regions, and scores of TFBSs using the DiffBind package.

Herein, *POU5F1B, ZNF662, ZNF442, KIN, ZNF410*, and *SGSM2* TFs were found in the *meta-analysis* of MCF7 cell lines treated with 10 nM E2 but not in individual studies. *POU5F1B,* a processed pseudogene highly homologous to *OCT4*, was recently shown to be transcribed in ER^+^ BC [57]. *POU5F1B* is specifically expressed in mammalian totipotent embryonic stem and germ cells and has a crucial role in regulating and maintaining pluripotency and self-renewal [57]. The Zinc finger protein family often functions in transcriptional regulation through sequence-specific DNA binding [58]. *ZNF662, ZNF442, and ZNF410* were involved in the regulation of transcription by RNA polymerase II [59]. It has shown that low expression of G protein-coupled estrogen receptor 1 (*GPER1*) is significantly associated with adverse survival of BC patients [60]. *ZNF662* was identified as one of the unique factors related to *GPER*-DNA binding [60]. *ZNF442* plays a role in the strategy adopted by ER^+^ BC and triple-negative breast cancer (TNBC) cell lines for maintaining zinc homeostasis [61]. *ZNF410* uniquely activates *CHD4*, one of the catalytic components of the Nucleosome Remodeling and Deacetylase (NuRD) complex [62]. NuRD has been shown to have opposing effects on cancer. For instance, promoting and inhibiting tumor growth and metastasis in different tissues such as ER^+^ BC [63, 64]. Recently, it has been shown that the Knockdown of DNA/RNA‑binding protein KIN17 (*KIN*) promotes apoptosis of TNBC [65]. Small G protein signaling modulator 2 (*SGSM2*) is involved in ER^+^ BC metastasis by enhancing migrator cell adhesion via interaction with E-cadherin [66].

With enrichment analysis of meta-DBSs-associated common genes by ChEA3, TFs including *PCGF2, HNF1B, and ZBED6* were predicted as regulatory factors involved in BC progression. Based on our findings, no peaks associated with those TFs were found in individual studies and *meta-analysis* result*s*. Maybe it is because all TFs do not always regulate their own expression. Polycomb group proteins (PcG) play a critical role in cancer development, proliferation, senescence, and carcinogenesis [67]. *PCGF2* serves as a tumor suppressor in BC, gastric cancer, and colon cancer, probably for the negative regulation of Akt activation [68]. Recent studies have shown that methylation of homeobox genes, such as the *HNF1B*, plays a critical role in BC's insurgence or progression [69]. Zinc Finger BED-Type Containing 6 (*ZBED6*) has been shown to repress insulin-like growth factor 2 (*IGF2*) transcription [59]. The role of *ZBED6* has only been reported in TNBC but is not well understood in ER^+^ BC so far [70].

Most TFs indicated in Fig. 4 were shared among results of individual studies, *meta-analysis*, and the top 50 TFs obtained from ChEA3 (Fig. 6). The *ESR1* and *PGR*, which play an essential role in the development of BC, were ranked as top-ranking TFs (Fig. 6). Because ChEA3 ranks TFs only based on a gene list without any assumptions, it confirms the correctness of our results. It verifies that removing batch effects and performing a correct *meta-analysis* can produce more exact results. *POLRMT* and *TFAM* are human mitochondrial TFs [71]. As an essential mitochondrial DNA (mtDNA)-binding protein, *TFAM* functions in genome maintenance such as determining the abundance of the mitochondrial genome. Emerging evidence indicates that altered *TFAM* levels or mtDNA copy numbers may impact mitochondrial homeostasis in Alzheimer's and other neurodegenerative diseases [71]. Several studies suggested that *TFAM* TFs are potential biomarkers for the prognosis of BC [72]. *POLRMT* is a mitochondrial RNA polymerase that requires *TFAM* for mitochondrial transcription initiation [73]. Targeting both those factors is a promising strategy for sensitizing BC cells to chemotherapy [72, 74]. Although most of the datasets analyzed in the present study have used the chip-anti-ER (Santa Cruz HC-20, cat# SC-543), the tagging approach could alter the binding preferences of the protein, and therefore the experimental results.

Interestingly, the significant GO terms and KEGG pathways obtained from PSFEA in the present study were enriched by many predicted TFs by ChEA3 (Figs. 6 and 7). Therefore, *meta-analysis* led to the recognition of TFs involved in the development of BC. Cornification (GO:0070268) [75], anterior/posterior pattern specification (GO:0009952) [76], embryonic organ morphogenesis (GO:0048562) [77], response to inorganic substance (GO:0010035) [78], and negative regulation of transcription by RNA polymerase II (GO:0000122) [79] have been previously reported in ER^+^ BC. Tissue development (GO:0009888) [80] was enriched in BC. Cortical actin cytoskeleton (GO:0030864) [81], and cortical cytoskeleton (GO:0030863) [81] in pancreatic cancer, and cytoskeleton (GO:0005856) [82, 83] in ovarian cancer were enriched but not reported in ER^+^ BC previously. Regulation of anatomical structure morphogenesis (GO:0022603) [84] in colorectal cancer and phosphatidylcholine metabolic process (GO:0046470) [85] in hepatocellular carcinoma were enriched. Response to metal ion (GO:0010038) [86] in chronic myeloid leukemia cells, and negative regulation of RNA biosynthetic process (GO:1902679) [87] in Alzheimer’s Disease, were enriched but not reported in BC before. Regulatory region nucleic acid binding (GO:0001067) [88], lipid binding (GO:0008289) [89], RNA polymerase II regulatory region DNA binding (GO:0001012) [90], activating transcription factor binding (GO:0033613) [91], DNA-binding transcription factor binding (GO:0140297) [92], transcription coregulator activity (GO:0003712) [93] have been previously reported in ER^+^ BC. Glucose binding (GO:0005536) [94] was enriched in multiple myeloma but not reported in BC before.

The nine enriched KEGG pathways include Pathways in cancer (hsa05200) [95], Estrogen signaling pathway (hsa04915) [95], Rap1 signaling pathway (hsa04015) [96], Neurotrophin signaling pathway (hsa04722) [97], and Fluid shear stress and atherosclerosis (hsa05418) [98] have been previously reported in ER^+^ BC. MAPK signaling pathway (hsa04010) [99] was enriched in MCF7 cell lines of the resistance to treatment. Tight junction (hsa04530) [100], and Bladder cancer (hsa05219) [101, 102] have already been reported tumor BC. A study has shown that Thyroid cancer (hsa05216) [103] is related to metabolic pathways which were enriched in TNBC.

According to the cases mentioned, GO terms and pathways previously approved concerning BC were enriched (Fig. 7). Moreover, many GO terms and pathways identified were associated with gastrointestinal cancers, including colorectal and hepatocellular carcinoma. In particular, our findings showed that many TFBSs associated with mitochondrial genes are directly affected by ER, including *POLRMT* and *TFAM* TFs. Especially *TFAM* increased expression has been reported in a *meta-analysis* of MCF7 cell lines treated with E2 [32]. This may clarify why the metabolism pathways were identified along with the estrogen signaling pathway in BC (Fig. 7 and see Additional file 2: Table S22). Although some relationships are found between BC and hepatocellular carcinoma, more studies are needed on these TFs and their associated pathways.

The comparison of ChIP-seq and RNA-seq data *meta-analysis* results showed that many TFs predicted by ChEA3 were up-regulated in RNA-seq *meta-analysis* results. There can be several reasons why some TFs are down-regulated. *FOXA1* is a determinant of drug resistance in breast cancer cells, and it has different functions in response to treatment in ER^+^ and TNBC cell lines [33] (Table 3). Investigation of primary regulatory regions has shown that there is plasticity in ER binding, with distinct ER binding profiles associated with clinical outcome. These differential ER binding profiles appear to be mediated by *FOXA1* [104].

*SPDEF* also is with both oncogenic and tumor-suppressor functions in BC [105]. Therefore, according to its role in the cell, it can have a different expression. Furthermore, the treatment period of RNA-seq data can be effective in the gene expressions, so it is possible that at one time, the expression of the gene is increased, and at another time, it is down-regulated.

Our study was constrained by the number of datasets with the same conditions. Although the results were remarkable and substantial, they could be more precise and reliable if more data were combined. In addition, despite generating large volumes of datasets related to BC in databases, the data with the same conditions is negligible. Therefore, developing datasets with more uniform and focused conditions is could help to detect more precise *meta-analysis* results.

## Conclusion

In the current study, we applied a workflow for integrating and performing *meta-analysis* of several ChIP-seq studies based on TFBSs scores in ER^+^ BC. The same number, regions, and scores of TFBSs were obtained for all samples. Then, the known and unknown batch effects were removed, and the *meta-analysis* was performed to obtain meta-DBSs. Some TFs were significantly identified in the *meta-analysis* but not in individual studies. Also, with meta-DBSs enrichment, many GO terms and pathways were identified that were not previously reported in BC. Finally, by enriching the meta-DBSs-associated genes, some TFs were prioritized and predicted as potential regulators. Those TFs were not found in the results of the *meta-analysis* and individual studies. Moreover, the results of the ChIP-seq *meta-analysis* were confirmed by comparing them with the *meta-analysis* of the RNA-seq data. The results showed that many TFs predicted by ChEA3 were up-regulated in RNA-seq *meta-analysis* or individual datasets results. As a suggestion, this workflow can be applied to other types of BC, such as HER2^+^ and other diseases, provided that there are sufficient datasets with the same conditions. It can also be performed on other TFs involved in BC that cooperate with ER as a cofactor, revealing new insights into ER mechanism of action.

### Supplementary Information

**Additional file 1: Figure S1.** (A) The Heat map Correlation matrix and (B) PCA plot for the GSE94023 study. MCF7 cell line was treated with E2 for different times, including 0 (treated with vehicle as control), 5, 10, 20, 40, 80, 160, 320, 640, and 1280 minutes. According to the matrix, after 40 minutes, E2-treated samples showed a more significant difference compared to the control sample. **Figure S2.** The Heat map Correlation matrix for GSE94023, GSE99626, GSE67295, and GSE115607 studies. The default binding affinity matrix was obtained from the DiffBind package. The datasets with MCF7 cell lines that were treated with 10nM E2 for 40 or 45 minutes were shown (A) before and (B) after normalization and unknown batch effect correction. Batch effect removal was performed using the ARSyNseq package in R. **Figure S3.** The PCA plot for GSE94023, GSE99626, GSE67295, and GSE115607 studies. The default binding affinity matrix was obtained from the DiffBind package. The datasets with MCF7 cell lines that were treated with 10nM E2 for 40 or 45 minutes were shown (A) before and (B) after normalization and unknown batch effect correction. **Figure S4.** The Heat map Correlation matrix for GSE23893, GSE54855, and GSE59530 studies. The default binding affinity matrix was obtained from the DiffBind package. The datasets with MCF7 cell lines that were treated with 100nM E2 for 40 or 45 minutes were shown (A) before and (B) after normalization and unknown batch effect correction. Batch effect removal was performed using the ARSyNseq package in R. **Figure S5.** The PCA plot for GSE23893, GSE54855, and GSE59530 studies. The default binding affinity matrix was obtained from the DiffBind package. The datasets with MCF7 cell lines that were treated with 100nM E2 for 40 or 45 minutes were shown (A) before and (B) after normalization and unknown batch effect correction.

**Additional file 2: Table S1.** Differentially bound sites (DBSs) obtained from MCF7 cell line treated with 10nM E2 for 45 minutes in GSE94023 study. **Table S2.** Differentially bound sites (DBSs) obtained from MCF7 cell line treated with 10nM E2 for 45 minutes in GSE99626 study. **Table S3.** Differentially bound sites (DBSs) obtained from MCF7 cell line treated with 10nM E2 for 45 minutes in GSE67295 study. **Table S4.** Differentially bound sites (DBSs) obtained from MCF7 cell line treated with 10nM E2 for 45 minutes in GSE115607 study. **Table S5.** Differentially bound sites (DBSs) obtained from T47D cell line treated with 10nM E2 for 45 minutes in GSE80367 study. **Table S6.** Differentially bound sites (DBSs) obtained from T47D cell line treated with 100nM E2 for 45 minutes in GSE23893 study. **Table S7.** Differentially bound sites (DBSs) obtained from MCF7 cell line treated with 100nM E2 for 45 minutes in GSE23893 study. **Table S8.** Differentially bound sites (DBSs) obtained from MCF7 cell line treated with 100nM E2 for 45 minutes in GSE54855 study. **Table S9. **Differentially bound sites (DBSs) obtained from MCF7 cell line treated with 100nM E2 for 45 minutes in GSE59530 study. **Table S10.** Default binding affinity matrix of 6 samples by the 63,612 sites that overlap in at least two of the samples using DiffBind in (GSE94023, GSE99626, GSE67295, & GSE115607) MCF7 cell line treated with 10nM E2 for 45 minutes. **Table** **S11.** Default binding affinity matrix of 6 samples by the 23,517 sites that overlap in at least two of the samples using DiffBind in (GSE23893, GSE54855, & GSE59530) MCF7 cell line treated with 100nM E2 for 45 minutes. **Table S12.** Meta-differentially bound sites (meta-DBSs) obtained from a meta-analysis on (GSE94023, GSE99626, GSE67295, & GSE115607) MCF7 cell line treated with 10nM E2 for 45 minutes. **Table S13.** Meta-differentially bound sites (meta-DBSs) obtained from a meta-analysis on (GSE23893, GSE54855, & GSE59530) MCF7 cell line treated with 100nM E2 for 45 minutes. **Table S14.** literature_ChIP-seq. **Table S15.** Enrichr. **Table S16.** ARCHS4—Coexpression. **Table S17.** ENCODE--ChIP-seq. **Table S18.** ReMap--ChIP-seq. **Table S19.** GTEx—Coexpression. **Table S20.** Integrated_topRank. **Table S21.** Integrated_meanRank. **Table S22.** Gene Ontology (GO) for 7,308 meta-DBSs related to 617 common genes among MCF7 & T47D cell lines using Cistrome-GO. **Table S23.** KEGG pathways analysis for 7,308 meta-DBSs related to 617 common genes among MCF7 & T47D cell lines using Cistrome-GO. **Table** **S24. **Differentially expressed genes (DEGs) identified from GRO-seq data in the MCF7 cell line treated with 100nM E2 for 40 minutes in the GSE27463 study.

## Data Availability

The datasets used in the current study including GSE94023, GSE99626, GSE67295, GSE115607, GSE80367, GSE23893, GSE54855, and GSE59530 are publicly available and were collected from SRA-NCBI (https://www.ncbi.nlm.nih.gov/) database. They were downloaded from ENA-EBI (https://www.ebi.ac.uk/ena/browser) database.

## Abbreviations

BC: Breast cancer
DEGs: Differentially expressed genes
ER^+^: Estrogen receptor positive
HER2^+^: Human epidermal growth factor receptor 2
GSEA: Gene set enrichment analysis
GO: Gene ontology
KEGG: Kyoto Encyclopedia of Genes and Genomes
Meta-DEGs: Mata-differentially expressed genes
TNBC: Triple negative breast cancer
TFBSs: Transcription factor binding sites
DBSs: Differentially bound sites
Meta-DBSs: Meta-differentially bound sites
PSFEA: Peak set functional enrichment analysis
ChEA: ChIP Enrichment Analysis
TSS: Transcription start site
