# Supplementary material for: Meta-analysis of integrated ChIP-seq and transcriptome data revealed genomic regions affected by estrogen receptor alpha in breast cancer
Source: BMC Med Genomics. 2023 Sep 15;16:219. doi: 10.1186/s12920-023-01655-z (PMC10503144; doi:10.1186/s12920-023-01655-z)
Supplement: Supplementary file 1 — Additional file 1: Figure S1. (A) The Heat map Correlation matrix and (B) PCA plot for the GSE94023 study. MCF7 cell line was treated with E2 for different times, including 0 (treated with vehicle as control), 5, 10, 20, 40, 80, 160, 320, 640, and 1280 minutes. According to the matrix, after 40 minutes, E2-treated samples showed a more significant difference compared to the control sample. Figure S2. The Heat map Correlation matrix for GSE94023, GSE99626, GSE67295, and GSE115607 studies. The default binding affinity matrix was obtained from the DiffBind package. The datasets with MCF7 cell lines that were treated with 10nM E2 for 40 or 45 minutes were shown (A) before and (B) after normalization and unknown batch effect correction. Batch effect removal was performed using the ARSyNseq package in R. Figure S3. The PCA plot for GSE94023, GSE99626, GSE67295, and GSE115607 studies. The default binding affinity matrix was obtained from the DiffBind package. The datasets with MCF7 cell lines that were treated with 10nM E2 for 40 or 45 minutes were shown (A) before and (B) after normalization and unknown batch effect correction. Figure S4. The Heat map Correlation matrix for GSE23893, GSE54855, and GSE59530 studies. The default binding affinity matrix was obtained from the DiffBind package. The datasets with MCF7 cell lines that were treated with 100nM E2 for 40 or 45 minutes were shown (A) before and (B) after normalization and unknown batch effect correction. Batch effect removal was performed using the ARSyNseq package in R. Figure S5. The PCA plot for GSE23893, GSE54855, and GSE59530 studies. The default binding affinity matrix was obtained from the DiffBind package. The datasets with MCF7 cell lines that were treated with 100nM E2 for 40 or 45 minutes were shown (A) before and (B) after normalization and unknown batch effect correction. [file 12920_2023_1655_MOESM1_ESM.docx]

**
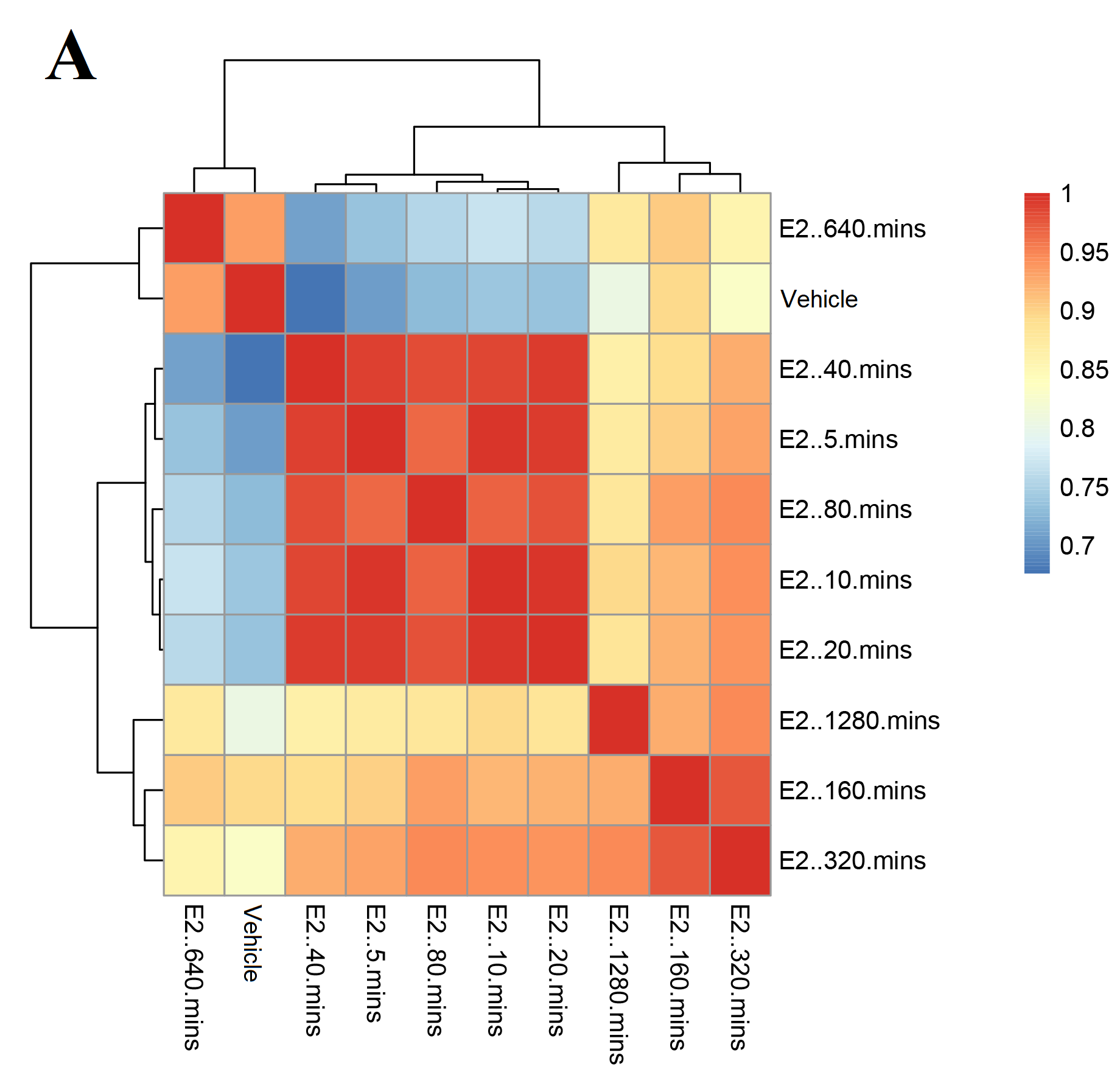
**


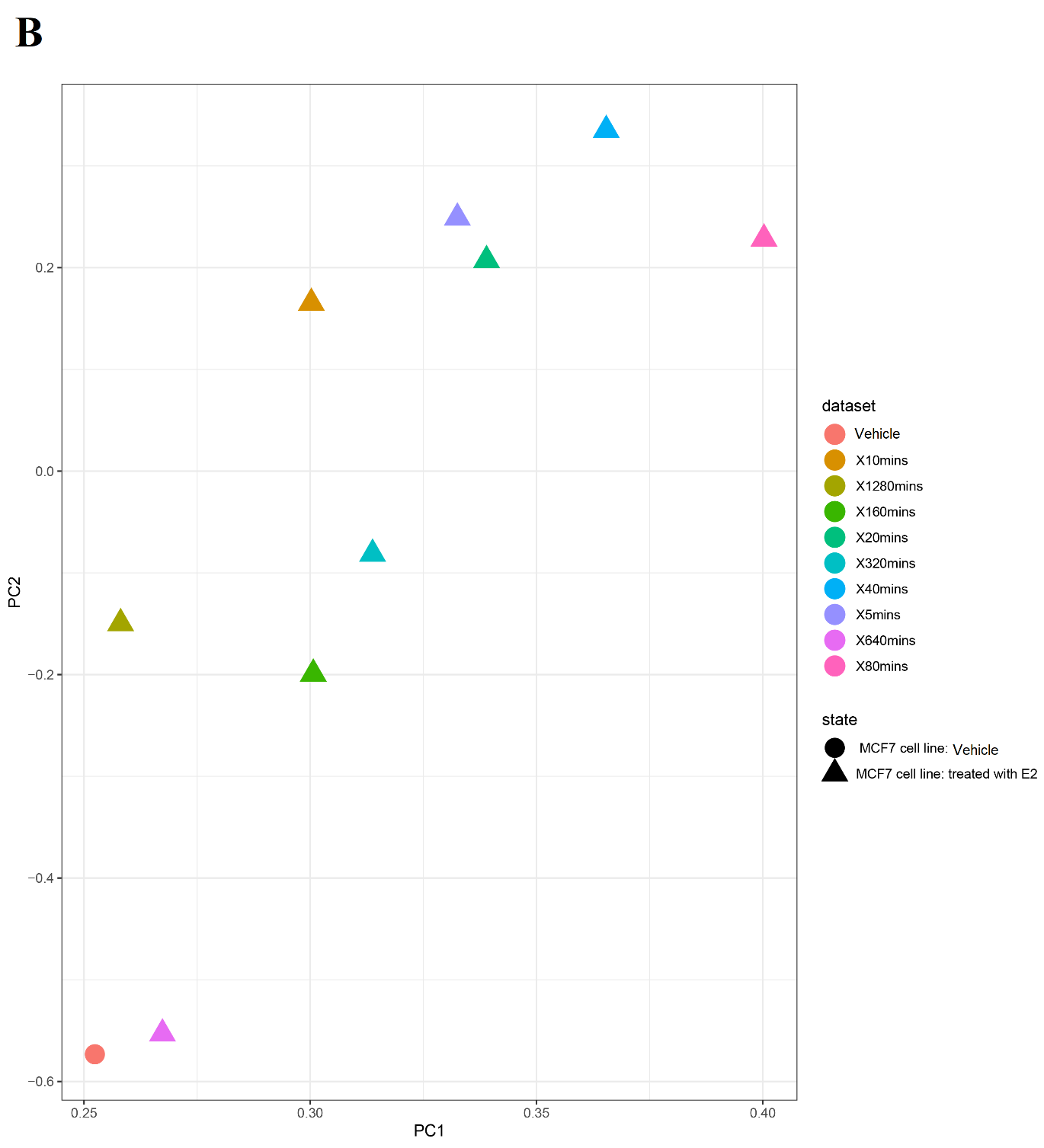


**Additional file 1: Figure S1. (A)** **The Heat map Correlation matrix and (B) PCA plot for the GSE94023 study.** MCF7 cell line was treated with E2 for different times, including 0 (treated with vehicle as control), 5, 10, 20, 40, 80, 160, 320, 640, and 1280 minutes. According to the matrix, after 40 minutes, E2-treated samples showed a more significant difference compared to the control sample.


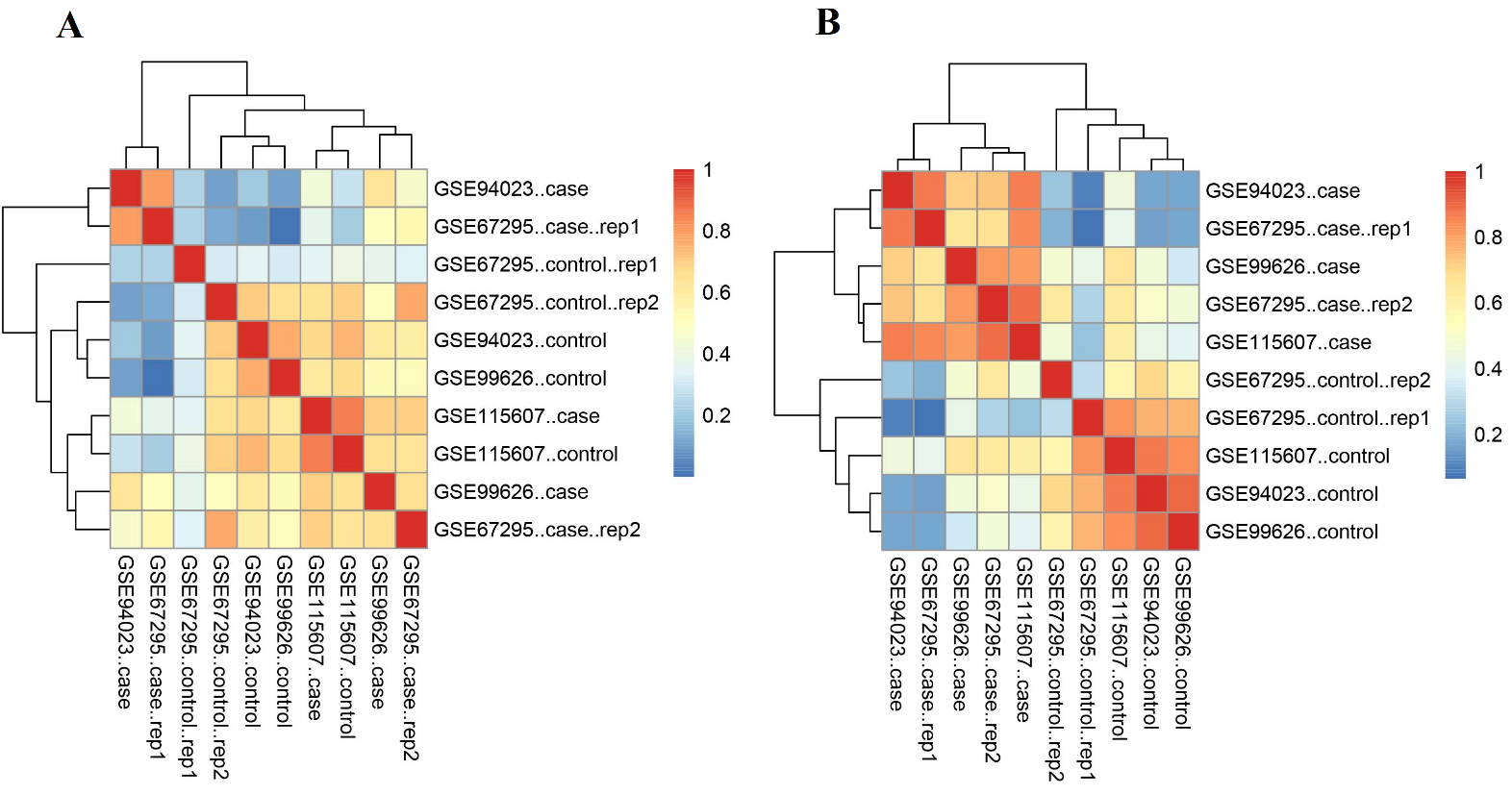


**Additional file 1: Figure S2.** **The Heat map Correlation matrix for GSE94023, GSE99626, GSE67295, and GSE115607 studies.** The default binding affinity matrix was obtained from the DiffBind package. The datasets with MCF7 cell lines that were treated with 10nM E2 for 40 or 45 minutes were shown (A) before and (B) after normalization and unknown batch effect correction. Batch effect removal was performed using the ARSyNseq package in R.


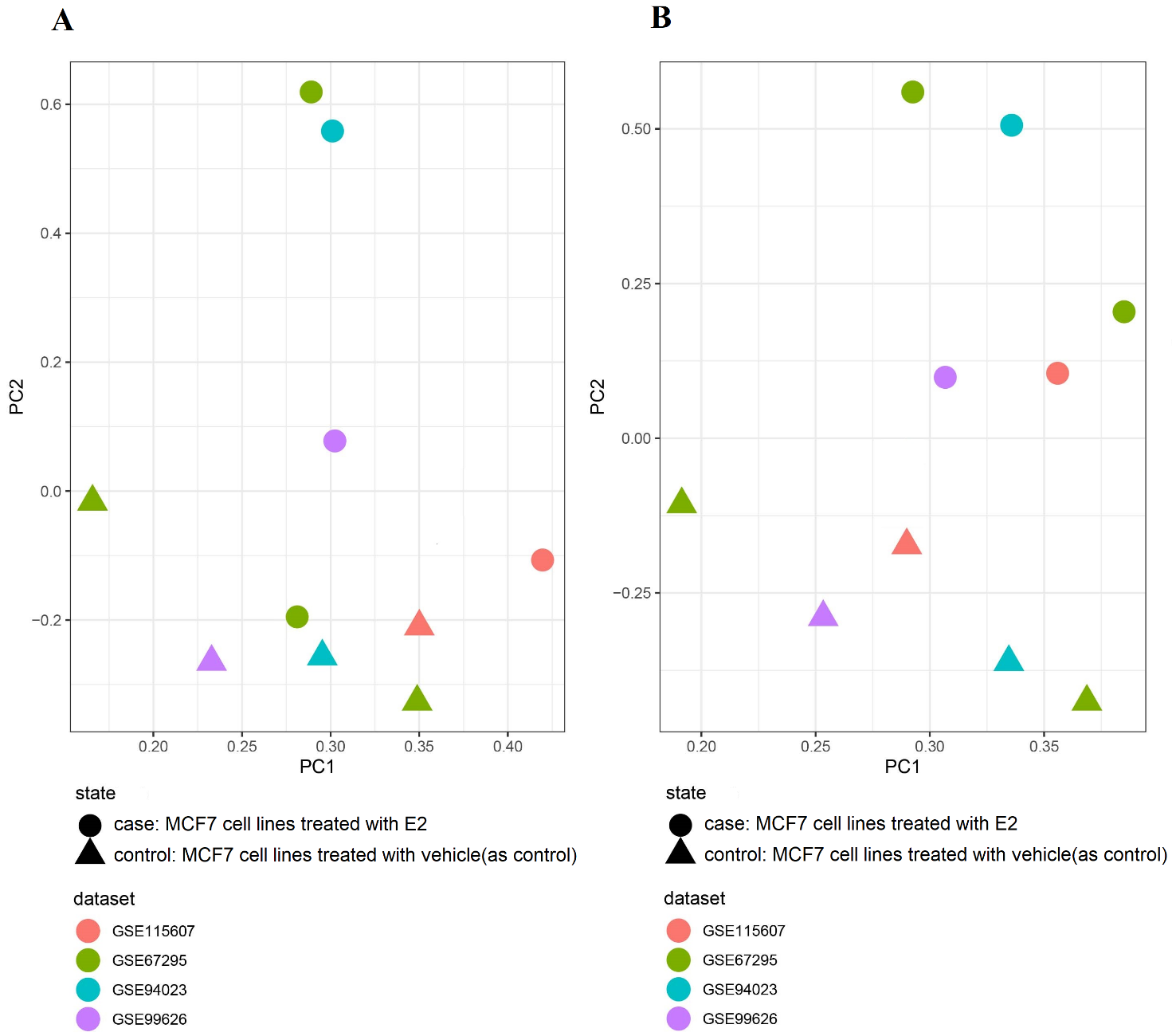


**Additional file 1: Figure S3. The PCA plot for GSE94023, GSE99626, GSE67295, and GSE115607 studies.** The default binding affinity matrix was obtained from the DiffBind package. The datasets with MCF7 cell lines that were treated with 10nM E2 for 40 or 45 minutes were shown (A) before and (B) after normalization and unknown batch effect correction.


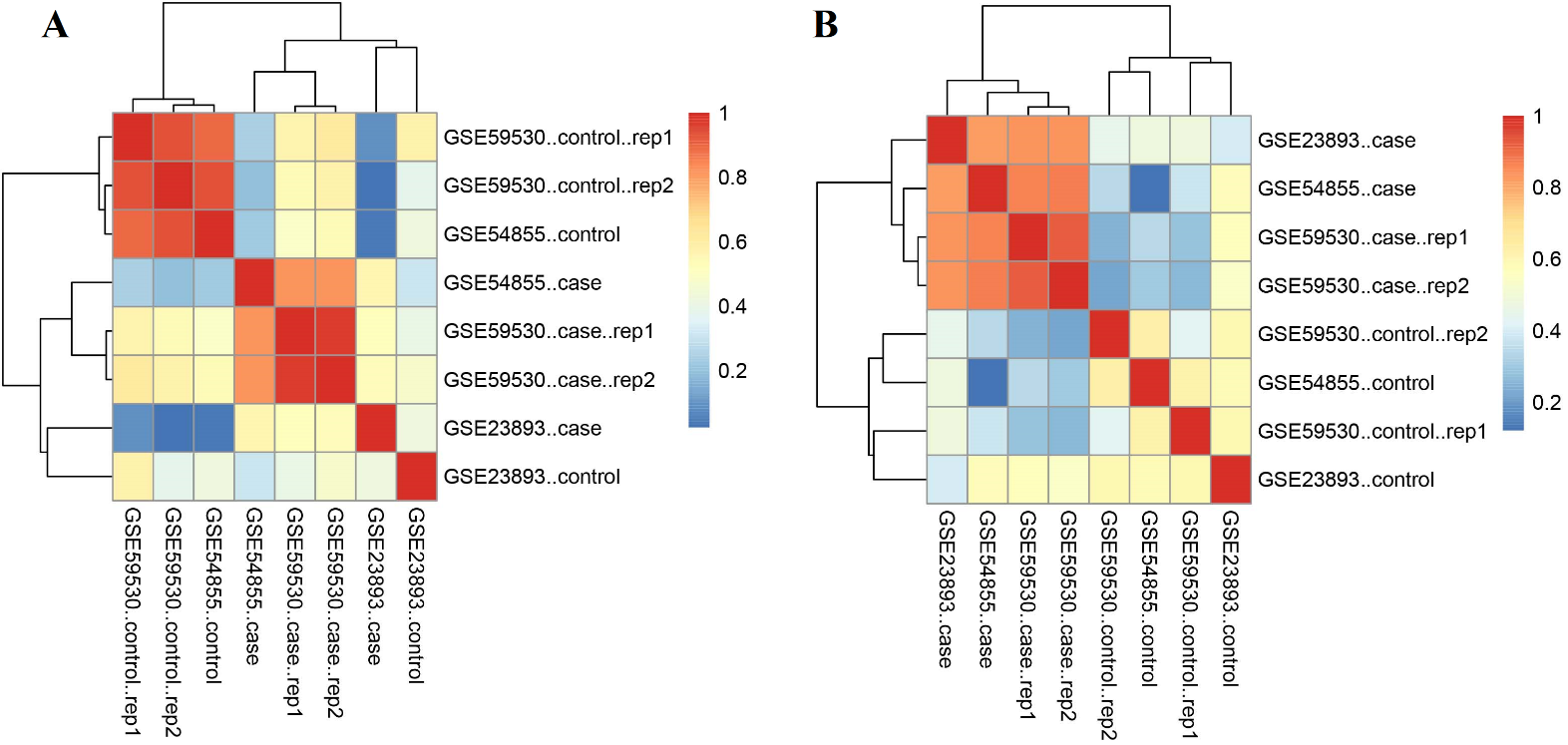


**Additional file 1: Figure S4.** **The Heat map Correlation matrix for GSE23893, GSE54855, and GSE59530 studies.** The default binding affinity matrix was obtained from the DiffBind package. The datasets with MCF7 cell lines that were treated with 100nM E2 for 40 or 45 minutes were shown (A) before and (B) after normalization and unknown batch effect correction. Batch effect removal was performed using the ARSyNseq package in R.


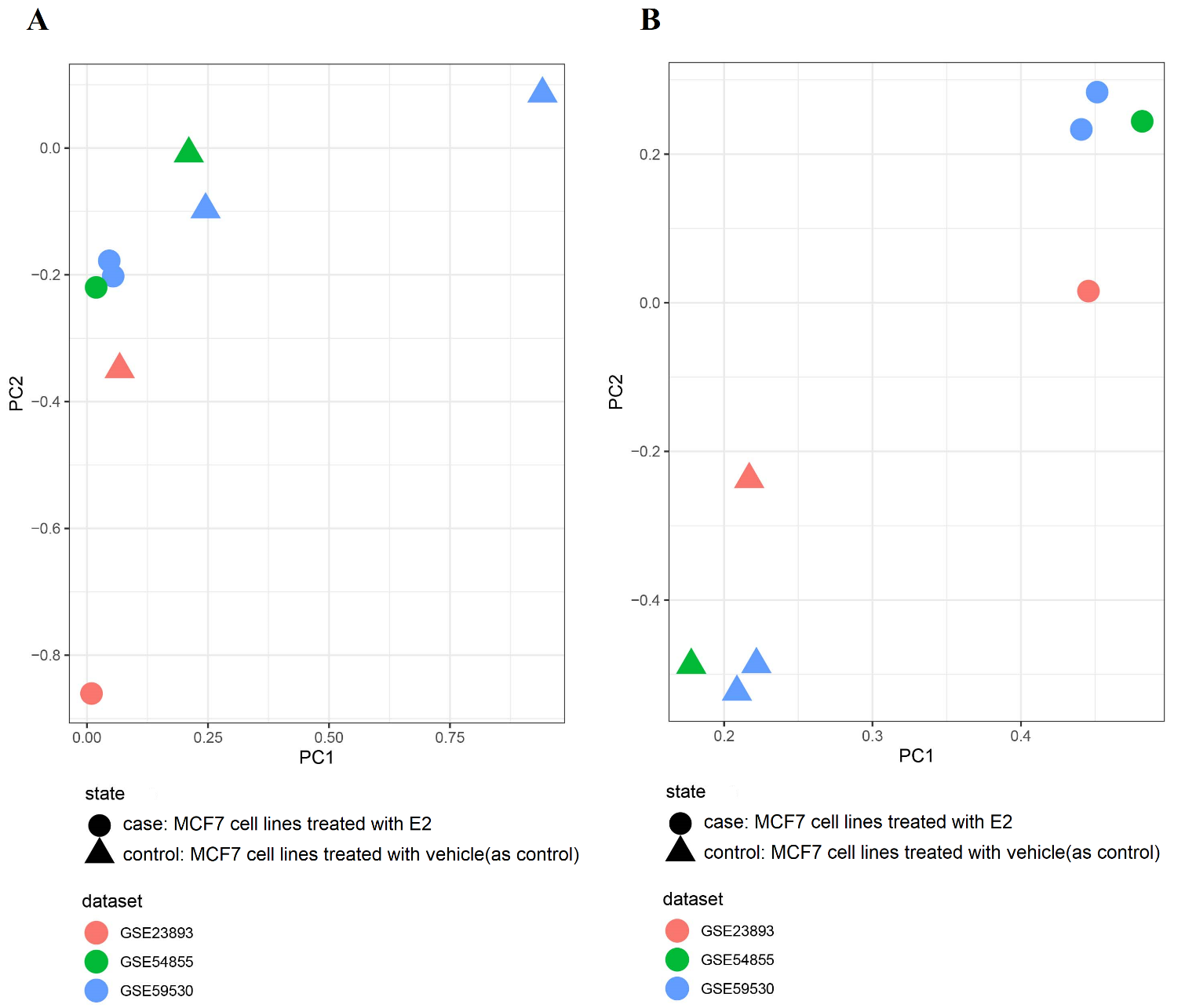


**Additional file 1: Figure S5. The PCA plot for GSE23893, GSE54855, and GSE59530 studies.** The default binding affinity matrix was obtained from the DiffBind package. The datasets with MCF7 cell lines that were treated with 100nM E2 for 40 or 45 minutes were shown (A) before and (B) after normalization and unknown batch effect correction.
